# Influence of Diamond-Like Carbon Coating on the Channel Deformation of Injection-Molded Microfluidic Chips during the Demolding Process

**DOI:** 10.3390/polym12122914

**Published:** 2020-12-04

**Authors:** Yilei Wang, Bingyan Jiang, Mingyong Zhou, Jiachen Chen, Can Weng

**Affiliations:** 1 College of Mechanical and Electrical Engineering, Central South University, Changsha 410083, China; wangyilei_csu@csu.edu.cn (Y.W.); mingyong.zhou1989@csu.edu.cn (M.Z.); csuchenjiachen@csu.edu.cn (J.C.); 2 State Key Laboratory of High Performance Complex Manufacturing, Central South University, Changsha 410083, China

**Keywords:** injection molding, microfluidic chip, diamond-like carbon coating, channel morphology, deformation

## Abstract

Injection molding is one of the main techniques for manufacturing microfluidic chips. As an important stage, the demolding process in injection molding will directly affect the quality of the functional unit of microfluidic chips (polymer microchannels), thus limiting the realization of its functions. In this study, molecular dynamics (MD) simulations on the demolding process were carried out to investigate the influence of diamond-like carbon (DLC) coating on the channel deformation. The channel qualities of polystyrene (PS), polymethyl methacrylate (PMMA), cyclic olefin copolymer (COC) and polycarbonate (PC) were analyzed after demolding with nickel (Ni) and DLC-coated mold inserts, respectively. In particular, the non-bonded interfacial interaction energy, elastic recovery and gyration radius of polymer molecular chains were further studied. The results showed that the non-bonded interfacial interaction energies could be significantly reduced by DLC-coating treatment on the mold insert. Moreover, common channel defects such as molecular chain separation, surface burrs and necking did not occur. The treatment of DLC coating could also significantly reduce the change in the gyration radius of polymer molecular chains, so the morphology of the polymer channel could be maintained well. However, the change in the elastic recovery of the polymer channel was increased, and the opening width became larger. In a word, DLC-coating treatment on the mold insert has great application potential for improving the demolding quality of injection-molded microfluidic chips.

## 1. Introduction

Microfluidic chips have been widely used in life science, chemical analysis, medical detection and other fields [1]. The main functional structure of the microfluidic chip is the microchannel, as to complete fluid filling, sample separation and detection. The performance of the microfluidic chip thus largely depends on the replication quality of these channels. Injection molding technology is one of the main methods for microfluidic chip manufacturing [2], which has the advantages of high precision, low cost, high efficiency, and short cycle [3]. Its process mainly includes four stages as the filling, the packing, the cooling and the demolding. The demolding process plays an important role in the high-quality replication of the microchannels.

During the demolding process, the main deformations of the channels are warping, stretching, and surface burr [4,5]. Studies have shown that most of the deformations are mainly caused by friction [6], adhesion [7] and the thermal contraction force [8]. The friction and adhesion at the interface between the polymer and mold insert are the fundamental limiting factors for the high-quality replication of products [9]. In terms of decreasing friction and adhesion, surface-coating technologies like self-assembled monolayer (SAM) coating, diamond-like carbon (DLC) coating and ceramic coating [10,11,12,13] have been proven to be effective methods to improve the surface properties of metal mold insert and reduce interfacial interactions [13,14]. Among them, DLC coating is widely used in the surface treatment of metal mold insert, which has a low friction coefficient, high wear resistance and corrosion resistance under the condition of non-lubrication [15,16]. Griffiths et al. [17] studied the influence of DLC coating on the replication quality of microstructures in micro-injection molding by comparing the untreated mold insert in experiments, which could effectively reduce the demolding resistance. By depositing a doped diamond-like coating on the surface of silicon (Si) mold insert, Saha et al. [18] studied the influence of uncoated and nitrogen (N)-DLC-coated mold inserts on the quality of microstructures during the thermal nanoimprint process. The experimental results showed that N-DLC coating effectively improved the performance of Si micro mold insert, and the deformations of fracture and surface burrs were not observed on the surface of COC microstructures after demolding. Lu et al. [19] found that the DLC coating could provide a lower friction surface for copper substrate in micro-friction experiments. However, when the product size is reduced to the nanoscale, the intermolecular and surface forces have a significant impact on the interfacial adhesion [20,21], the experimental studies cannot directly analyze the internal mechanism of microstructures during the demolding process.

Thus, it is very important to further study the interfacial interactions in the fabrication of polymer microstructures from the molecular or atomic level. Molecular dynamics (MD) simulation methods for the analysis of interfacial interactions have been widely applied in nanoimprint lithography (NIL) [22,23], interfacial mechanisms [24,25], polymer injection molding [26,27], pulling simulations in diverse molecular systems [28,29] and other fields, which can accurately analyze the interaction mechanism of polymer-mold insert during the polymer molding process. Hsu et al. [30,31] used the MD method to study the influences of temperature, the aspect ratio of the stamp, and the anti-sticking layer on the polymer molding quality in the NIL process. Yang et al. [32] studied the interfacial adhesion characteristics of PMMA and the influence of the anti-sticking layer on the interfacial interaction in the NIL process by MD simulation. Wang et al. [33] studied the MD model of the tribochemical reaction between DLC coating and alumina (Al) and found that friction coefficient first increased and then decreased with the increase of temperature. Sun et al. [34] used MD simulation to study the influence of geometric morphologies of DLC-coated substrate on the adsorption behavior of potassium stearate monolayer. For micro-injection molding technology, Weng et al. [35,36] studied the influence of nanostructure shape, mold insert material and aspect ratio on the demolding process by MD simulation. Liu et al. [20] established an MD model of the polyphenylene sulfide (PPS)/Al heterogeneous interface to study the polymer–metal interaction in nano-injection molding. Zhou et al. [37] studied the interaction between polymer and mold insert with different aspect ratios using the MD simulation method. Lai et al. [29] investigated the interfacial shear between osteopontin (OPN) and hydroxyapatite (HA) mineral layers with surface nanostructures by MD simulation. Gotzias et al. [38] conducted pulling simulations on pairs of different carbon nanotubes and computed the optimal distance between tubes as they were arranged in bundle configurations. However, there have been no reports about the influence of DLC coating on the channel deformation of microfluidic chips during the demolding process of injection molding through the MD method.

In this study, the MD method was used to simulate the channel deformation of a microfluidic chip during the demolding process of injection molding with an aspect ratio of 1:3 on Ni and DLC-coated mold inserts, respectively. The changes of interfacial interaction energy, elastic recovery, gyration radius of polymer molecular chains and the influence of DLC coating on the demolding quality were studied by analyzing the channel morphologies at different demolding moments. Furthermore, the mechanism of the channel deformation of a microfluidic chip was revealed to provide valuable theoretical guidance for improving the demolding quality of the microchannel.

## 2. Materials and Methods

### 2.1. Polymer Model Construction

Polymers with good thermoplastic properties, such as polystyrene (PS), cyclic olefin copolymer (COC), polycarbonate (PC), polymethyl methacrylate (PMMA) were selected as research materials. Taking polymer PS as an example, Materials Studio (MS) 7.0 was used to construct a PS mono-molecule chain with a polymerization degree of 20. The polymerization degree of the polymer molecular chain must be able to represent the minimum number of repeating units of this polymer and satisfy the slippage and disentanglement of the molecular chain during the demolding process. Smart Minimizer method was used to minimize the energy, and then 200 steps annealing was carried out. The amorphous unit of PS polymer was constructed by an amorphous cell module with 75 chains and the initial density of 1.05 g/cm^3^. A constant particle number, volume and temperature (NVT) ensemble cycling annealing, energy minimization and high-temperature relaxation were successively carried out to reduce the internal stresses of amorphous polymer system and obtain a structurally stable polymer model. Finally, the temperature of the polymer was heated to 570 K (297 °C) to obtain a completely molten polymer system. The PMMA, COC and PC models were established in a similar way, as the specific construction parameters show in Table 1.

### 2.2. Mold Models Construction

Nickel (Ni) and DLC-coated mold inserts were constructed in the simulations, respectively. Ni unit cell was imported from MS7.0 as the mold insert material, and the (1 0 0) surface of the unit cell was cut. The supercell structure of Ni cells was obtained by extending the (1 0 0) surface to about 4.5 (x) × 11.0 (y) × 3.0 (z) nm^3^. For the DLC-coated mold insert, a rapid liquid quenching method was used to construct an amorphous diamond-like carbon supercell structure [39,40,41]. First, a supercell structure of diamond was established at an initial temperature of 300 K. The system containing carbon atoms was heated to 9000 K to obtain the molten state, and then quenched the whole system to 300 K within 3.0 ps. After a period of preparation, an amorphous DLC model with the same size as the Ni mold insert was obtained. The density of DLC-coated mold insert was 2.69 g/cm^3^, and it was found in experiments that the density range was 1.8 g/cm^3^ to 3.4 g/cm^3^ [42]. A nanostructure with an aspect ratio of 1:3 (the depth of 1.5 nm and a width of 4.5 nm) was obtained by deleting the corresponding atoms in the specific region.

**Figure 1:**
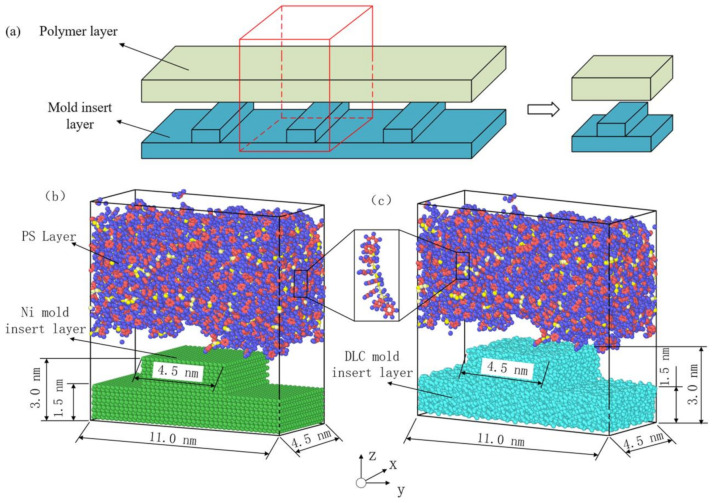
The molecular model in molecular dynamics (MD) simulation for the micro-injection molding process: (**a**) Representative model of actual injection molding process; (**b**) Ni–polystyrene (PS) system; (**c**) diamond-like carbon (DLC)–PS system.

Taking polymer PS as an example, an injection molding model was established with the polymer in the upper layer, and the rigid mold insert in the lower layer, as shown in Figure 1. For the shrinkage of polymer materials during the injection molding process, the x- and y-directions were set as periodic boundaries, and the z-direction was set as a non-periodic boundary condition.

### 2.3. Intermolecular Interaction Potential

The energy of the DLC-coated mold insert layer can be calculated by the Tersoff potential [40], expressed as Equations (1) and (2):

(1) $$E_{t}=\frac{1}{2}\underset{i}{\sum }\underset{j\neq i}{\sum }V_{ij}$$

(2) $$V_{ij}=f_{C}\left(r_{ij}\right)\left[f_{R}\left(r_{ij}\right)+b_{ij}f_{A}\left(r_{ij}\right)\right]$$

where *E_t_* is the energy of the DLC-coated mold insert layer, and $V_{ij}$ is the bond energy between atom i and atom j. $r_{ij}$ is the distance from atom i to atom j. The functions $f_{R}$ and $f_{A}$ represent a repulsive potential and an attractive potential, respectively. The function $f_{C}$ is a smooth cutoff function, and $b_{ij}$ is the bond angle term.

In this study, the polymer consistent force field (PCFF) was adopted to describe the intermolecular and non-bonding interfacial interactions between the atoms of the polymer layer [43]. The Van der Waals energy and electrostatic interaction energy between the polymer and the interface of mold insert can be calculated by 12–6 Lennard-Jones potential and Coulomb interaction potential [44], shown as Equation (3). Moreover, the interaction energies between different polymers and the interface of mold inserts can be approximated as adhesion energies [45], which are calculated by Equation (4):

(3) $$E_{non-bonded}=E_{vdw}+E_{ele}=4\varepsilon [{\left(\frac{\sigma }{r_{ij}}\right)}^{12}-(\frac{\sigma }{r_{ij}}{)}^{6}]+\frac{q_{i}q_{j}}{r_{ij}}(r_{ij}<r_{c})$$

where ε is a non-bonded interaction constant, σ is the distance between two atoms in equilibrium, $r_{ij}$ is the distance between two atoms at any time. $q_{i}$ and $q_{j}$ are the charge of atom i and atom j, respectively. $r_{c}$ is the cutoff distance, the Lennard-Jones potential and Coulomb interaction energies can be neglected when the distance between two atoms is out of the cutoff distance [36].

(4) $$E_{adhesion}=E_{\mathrm{int}eraction}=E_{total}-\left(E_{polymer}+E_{\mathrm{mold}}\right)$$

where $E_{adhesion}$, $E_{total}$, $E_{polymer}$ and $E_{\mathrm{mold}}$ are the interfacial interaction energy, the total energy of the polymer-mold system, the surface energy of the polymer without mold, and the surface energy of the mold without polymer, respectively.

### 2.4. Simulation Procedure

Anderson temperature control method and velocity Verlet time integration algorithm method were adopted to keep the mold insert temperature at a constant temperature of 393 K (120 °C). The entire filling and demolding process was accomplished through the large-scale atomic/molecular massively parallel simulator (LAMMPS), an open-source MD package in a computer cluster [46]. The filling process was completed within 50,000 fs during the injection molding process, and the package time was set to 20,000 fs. The models with fully filling and packing were selected as the initial models for the demolding process. Then the whole system was further cooled to 350 K (77 °C), below the glass transition (T_g_) temperature of the polymers. The external demolding force of 1.0 kcal/mol·Å was applied to each atom of the polymers along the z-direction. In order to effectively improve the calculation accuracy, the total number of steps was set to 70,000 with a time step of 0.05 fs during the demolding process with 1.25 nm cutoff distance, 0.30 nm spline width and 0.10 nm buffer width. The entire simulation process was carried out with the NVT ensemble. Both Van der Waals and Coulomb were calculated by the atom-based method during the simulation process.

## 3. Results and Discussions

### 3.1. Simulation of the Demolding Process

**Figure 2:**
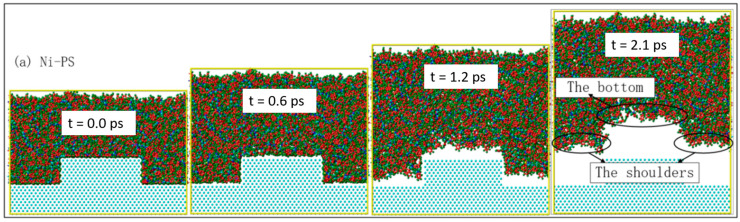

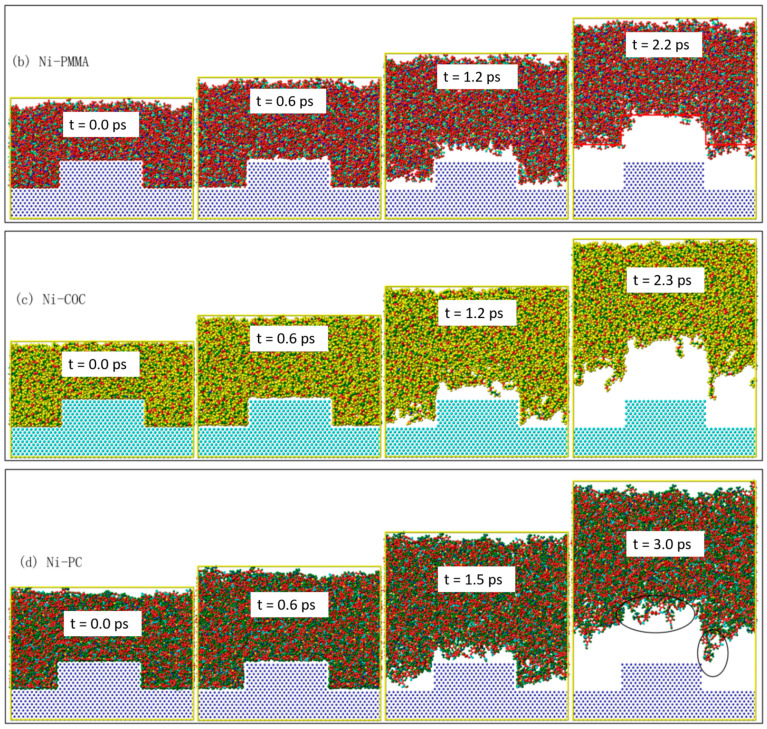
Snapshots of the demolding process of (**a**) PS, (**b**) polymethyl methacrylate (PMMA), (**c**) cyclic olefin copolymer (COC) and (**d**) polycarbonate (PC) on the Ni mold insert in MD simulation.

Figure 2 demonstrates the snapshots of four polymer channels during the demolding process on the Ni mold insert. At 0.0 ps, the four polymer materials were in a fully filled state. Due to the larger, the contact area between polymer and mold insert, the stronger the interfacial interaction [37]. There was no obvious separation between the polymer channel and mold insert at 0.6 ps, but separation at the bottom of the polymer channels occurred. With the unchanged morphology of the polymer channel, the polymer layer structure was stretched and elongated along the z-direction under the external demolding force. The shoulders and bottoms of the PS and PMMA channels were separated from the Ni mold insert at 1.2 ps. While the COC channels showed obvious deformation and failure, its molecular chains at the shoulders and bottom were seriously damaged. Slight surface burrs were found at the bottom of the PC channel. After demolding, PS, COC and PMMA were separated from the Ni mold insert at 2.1 ps, 2.3 ps and 2.2 ps, respectively. At this point, the PC was not completely separated until 3.0 ps. The molecular chains at the shoulders and bottom were most severely stretched along the z-direction for the demolding deformation of the PC channel. The deformations of the COC channel mainly included stretched molecular chains, elongated structure and serious surface burrs [26]. While the PS and PMMA channels with good demolding quality basically remained their geometrical shapes after demolding, as shown in Figure 2a,b.

**Figure 3:**
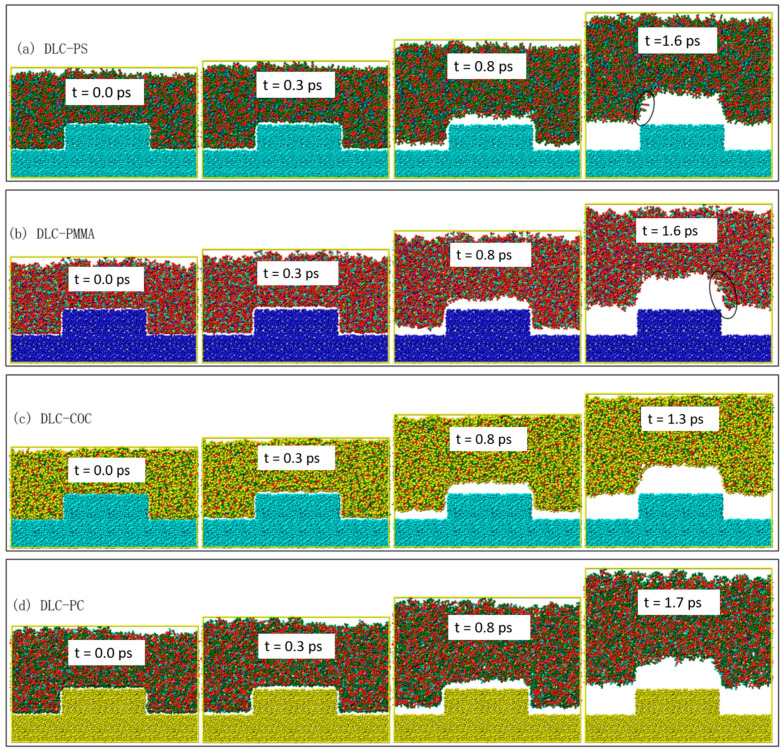
Snapshots of the demolding process of (**a**) PS, (**b**) PMMA, (**c**) COC and (**d**) PC on the DLC-coated mold insert in MD simulation.

In order to study the influence of DLC coating on the demolding quality of injection-molded microfluidic chips, the demolding process of four polymer channels on the Ni mold insert treated with DLC coating was simulated by the MD simulation method, as shown in Figure 3. Compared with the Ni mold insert on the demolding process, under the same external demolding force, the separation time of the polymer channels was significantly reduced, and the separation speed increased with the DLC-coated mold insert. At 0.0 ps, the four polymer materials were also in a fully filled state. The shoulders and bottoms of the polymer channels began to separate from the DLC-coated mold insert at 0.3 ps. At 0.8 ps, the shoulders of the polymer channels had been completely separated from the DLC-coated mold insert. At this time, the morphologies of these channels were maintained well without molecular chains stretching and surface burrs. The COC channels completed the entire demolding process at 1.3 ps. The PS, PMMA and PC channels were separated from the DLC-coated mold insert at 1.6 ps, 1.6 ps and 1.7 ps, respectively. The polymer channels could keep most of their original morphologies after demolding, especially COC molecular chains that were not separated and seriously stretched. The PS and PC channels had slight surface burrs after demolding. Due to the internal stresses of polymers, the opening width of the polymer channel was deformed to a certain extent compared with the design.

### 3.2. Non-bonded Interfacial Interaction Energy

**Figure 4:**
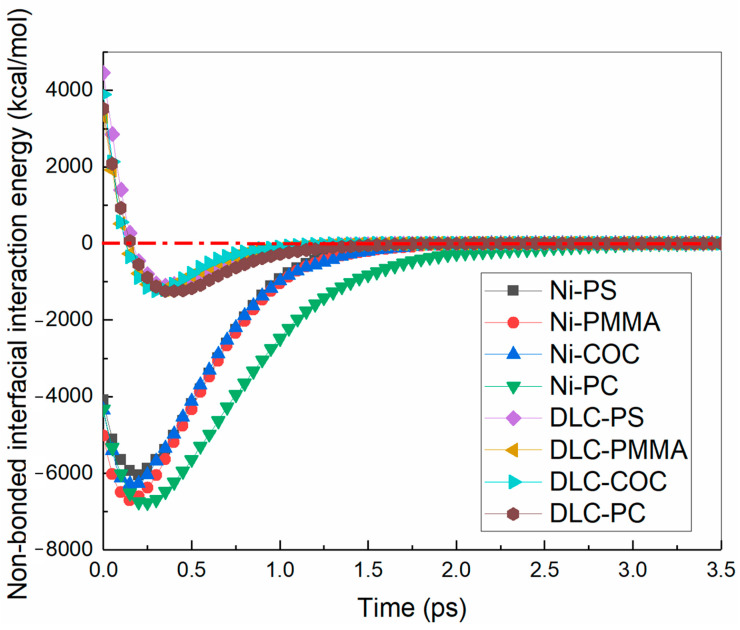
Non-bonded interfacial interaction energies of four polymer channels with the Ni and DLC-coated mold inserts.

To further study the internal mechanism of the polymer channel deformations of microfluidic chips on the Ni and DLC-coated mold inserts, the non-bonded interfacial interaction energies between polymers and mold inserts were calculated, as shown in Figure 4. Along z-direction was selected as the positive direction. The positive and negative values mentioned in this paper only represented the direction of the interfacial interaction energy, and their absolute values were expressed as the magnitude of the energy. When the mold insert was Ni, the non-bonded interfacial interaction energy was negative [35,36], indicating that there was adhesion. At the beginning of the demolding process, the Van der Waals force and the Coulomb force increased as the polymer molecular chains were pulled. Moreover, then the non-bonded interfacial interaction energy increased gradually. When the distance between polymer molecule chains at the shoulders and bottom of the channel and mold insert atoms increased, the non-bonded interfacial interaction energy began to decrease. At this point, the interfacial interaction between polymer molecular chains at the sidewall of the channel and mold insert atoms played a dominant role.

At about 0.2 ps, the non-bonded interfacial interaction energies of PS, PMMA, COC, and PC all reached their peak values with the Ni mold insert. Moreover, the peak value of the PC channel was the largest during the demolding process, which was −6762.68 kcal/mol. In contrast to the Ni mold insert, there was a repulsive force between polymer molecules and nonmetallic carbon atoms in the DLC-coated mold insert, and the non-bonded interfacial interaction energy was positive. After packing, the distance between polymer molecules and the DLC-coated mold insert atoms was minimum, the repulsive force and the non-bonded interfacial interaction energy reached the maximum. The peak values of non-bonded interfacial interaction energy of PS, PMMA, COC and PC channels were 4454.35 kcal/mol, 3320.40 kcal/mol, 3895.60 kcal/mol and 3521.65 kcal/mol with the DLC-coated mold insert, respectively. During the demolding process, the distances between polymer molecules and mold insert atoms and the non-bonded interfacial interaction energies were gradually increased. At about 0.2 ps, the non-bonded interfacial interaction energy of four polymer channels decreased to zero at first, and then increased gradually and reached the maximum at about 0.3 ps. This phenomenon indicated that polymer molecular chains were constantly stretched. During the demolding process with different mold inserts, the changes in non-bonded interfacial interaction energy of PS, PMMA, COC, PC channels were basically the same, regardless of the type of polymer materials. It was worth noting that the non-bonded interfacial interaction energies of four polymer channels were smaller with the DLC-coated mold insert, and the demolding speeds were faster than that with the Ni mold insert under the same external demolding force.

### 3.3. Elastic Recovery

**Figure 5:**
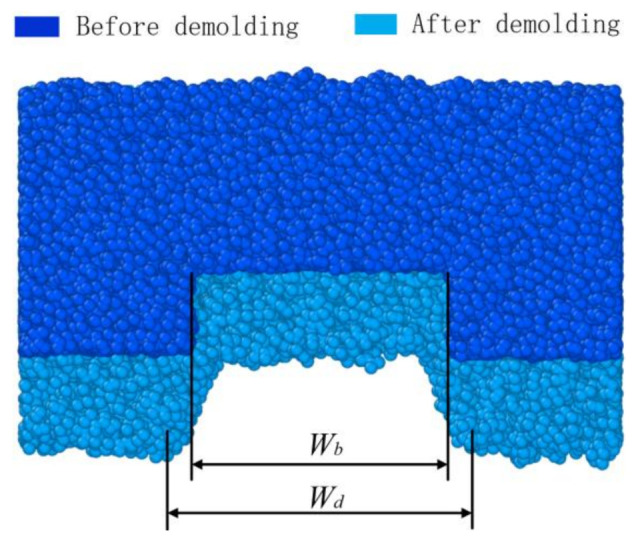
The widths of the polymer channels along y-direction before and after demolding.

**Figure 6:**
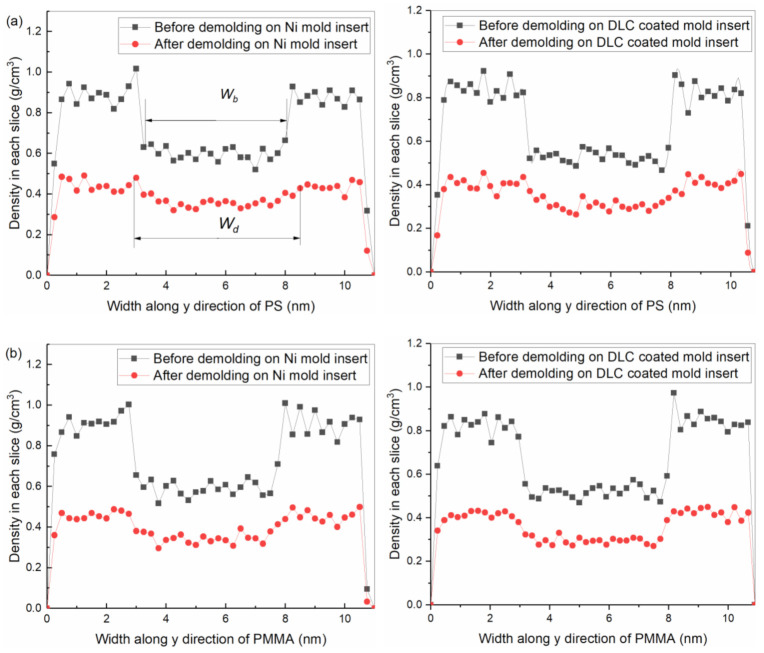

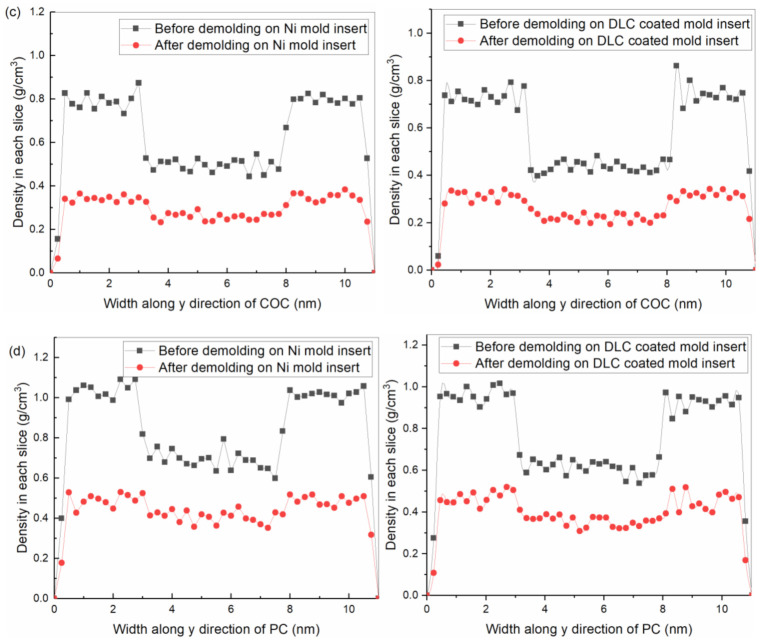
Densities in each slice of (**a**) PS, (**b**) PMMA, (**c**) COC and (**d**) PC channels before and after demolding with (left) the Ni and (right) DLC-coated mold inserts.

The channel size of the microfluidic chip is one of the key factors affecting its function; among them, the channel width is an important indicator of the molding quality [47]. Therefore, we need to investigate the width of the polymer channel before and after demolding. Due to the thermal shrinkage of the polymer itself, there was an elastic recovery of the polymer channel after demolding. The morphology of the polymer channel had a certain change. The widths before and after demolding were defined as *W_b_* and *W_d_*, respectively, as shown in Figure 5. They were calculated according to the first minimum density of each slice at the channel shoulders by using layer density along the y-direction with a slice thickness of 0.25 nm. The difference between channel width before and after demolding was defined as the change in elastic recovery. It is shown in Figure 6 that different polymer channel densities along the y-direction were calculated during the demolding process.

The changes in elastic recovery of PS, PMMA, COC, and PC channels along the y-direction were calculated, as shown in Table 2. After demolding, the density in each slice of the polymer structure significantly decreased. The surface morphologies of four polymer channels on the DLC-coated mold insert were better than that on the Ni mold insert. Due to the stretched molecular chain and loose structure, the maximum width of the COC channel was 0.40 nm after demolding on the Ni mold insert. The DLC coating treatment on mold insert could significantly solve the problems of voids and surface burrs of the polymer channels, though the change in the elastic recovery of the polymer channel was increased [31], and the opening width of the polymer channel became larger.

### 3.4. Radius of Gyration

The volume of the entire system was gradually changed during the demolding process. To further study the density change of polymer structure, the gyration radius (R_g_) was used to describe the extent of molecular chains extension in space [22], which was calculated by Equation (5):

(5) $$R_{g}{}^{2}=\frac{1}{M}\underset{i}{\sum }m_{i}{\left(r_{i}-r_{cm}\right)}^{2}$$

where M was the total mass of each polymer, $m_{i}$ was the atomic weight, $r_{i}$ and $r_{cm}$ were the distances of the atom to the center of mass position and the center of mass position of each polymer, respectively.

**Figure 7:**
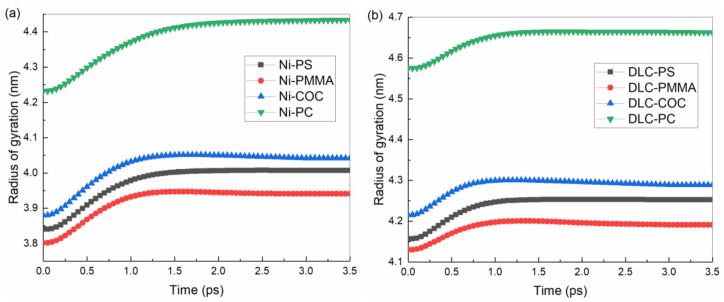
Gyration radii of molecular chains of four polymers on (**a**) the Ni and (**b**) DLC-coated mold inserts.

The variations in the gyration radius of polymer molecular chains during the demolding process of the entire system on the Ni and DLC-coated mold inserts were calculated, as shown in Figure 7. Before demolding, the system was fully filled with the minimum gyration radius of polymer molecular chains. Under the action of external demolding force, the gyration radius of the polymer structure gradually increased, but the increasing rate of the gyration radius decreased with the decrease of non-bonded interfacial interaction energy. Moreover, the rate finally tended to be stable [35,44].

After demolding, the changes in the gyration radius of molecular chains of PS, PMMA, COC, and PC channels were calculated, as shown in Table 3. Compared with the Ni mold insert in the demolding process, the changes in the gyration radius of molecular chains were significantly reduced on the DLC-coated mold insert. It indicated that the elongation of the entire polymer structure decreased. The demolding qualities of COC and PC channels were obviously improved. Their changes in the gyration radius of polymer molecular chains were 0.24 nm and 0.20 nm on the Ni mold insert, respectively. By contrast, their changes were only 0.07 nm and 0.09 nm on the DLC-coated mold insert. Due to the compact structure of PMMA and the high density of cohesive energy between molecule chains, the change in the gyration radius of polymer molecular chains was minimum after demolding. The molecular chains of polymer were continuously stretched in the influence of non-bonded interfacial interaction energy during the demolding process. After demolding, the gyration radii of four polymer molecular chains increased, and the polymer structures were elongated, which effectively explained the density change of the entire system.

## 4. Conclusions

In this study, the MD method was used to simulate the demolding process of polymer PS, PMMA, COC and PC on the Ni and DLC-coated mold inserts. The non-bonded interfacial interaction energy, elastic recovery and gyration radius were analyzed to find out the influence of DLC coating on improving the demolding quality of the polymer channel under the external demolding force of 1.0 kcal/mol·Å. The results showed that the quality of the polymer channels could be improved when the demolding process was implemented on the DLC-coated mold insert. There was no stretch of polymer molecular chains and serious surface burrs, which usually occur with the Ni mold inserts. Compared with the Ni mold insert, the peak values of non-bonded interfacial interaction energy could be significantly decreased with the DLC-coated mold insert, which were 4454.35 kcal/mol, 3320.40 kcal/mol, 3895.60 kcal/mol and 3521.65 kcal/mol for PS, PMMA, COC and PC channels, respectively. Additionally, the separation time between the polymer channel and the mold insert was obviously reduced, indicating that DLC coating had a positive effect on reducing the non-bonded interfacial interaction energy. The slice densities of the polymer channels were reduced similarly on the Ni and DLC-coated mold inserts. The opening width of the channel increased to a certain extent. The changes in the elastic recovery of PS, PMMA, COC and PC channels were calculated as 0.75 nm, 0.80 nm, 0.75 nm, 0.80 nm, respectively. The gyration radii of polymer molecular chains gradually increased on different mold inserts in the demolding progress. Consequently, a large change in the gyration radius indicated that a serious demolding deformation was caused. Moreover, the findings in this paper are indicative of the demolding process, and more rigorous simulation from the aspects of simulation methods, external demolding force and simulation time will be carried out in future work.

## Figures and Tables

**Table 1:** Construction parameters of four polymers.

Polymer	Polymerization Degree	Initial Temperature (K)	Total Number of Chains	Density (g/cm^3^)	Annealing Temperature (K)	Annealing Time (fs)
PS	20	300	75	1.05	570	500
COC	20	300	75	1.02	553	500
PC	20	300	37	1.20	570	500
PMMA	20	300	75	1.18	500	500

**Table 2:** Elastic recovery of four polymer channels along the y-direction.

Mold-Polymer Structure	*W_b_* (nm)	*W_d_* (nm)	Change in the Elastic Recovery (nm)
Ni-PS	5.00	5.25	0.25
Ni-PMMA	4.70	5.00	0.30
Ni-COC	4.75	5.15	0.40
Ni-PC	4.75	5.00	0.25
DLC-PS	5.00	5.75	0.75
DLC-PMMA	4.70	5.50	0.80
DLC-COC	4.75	5.50	0.75
DLC-PC	4.75	5.55	0.80

**Table 3:** Changes in the gyration radius of molecular chains of four polymers after demolding on different mold inserts.

Mold-Polymer Structure	Before Demolding (nm)	After Demolding (nm)	Change in the Gyration Radius (nm)
Ni-PS	3.84	4.01	0.17
Ni-PMMA	3.80	3.94	0.14
Ni-COC	3.80	4.04	0.24
Ni-PC	4.23	4.43	0.20
DLC-PS	4.16	4.25	0.09
DLC-PMMA	4.13	4.20	0.06
DLC-COC	4.22	4.29	0.07
DLC-PC	4.57	4.66	0.09
